# Harnessing the Neuroprotective Behaviors of Müller Glia for Retinal Repair

**DOI:** 10.31083/j.fbl2706169

**Published:** 2022-05-30

**Authors:** Juan S. Peña, Maribel Vazquez

**Affiliations:** 1 Department of Biomedical Engineering, Rutgers, The State University of New Jersey, Piscataway (08854), New Jersey, USA

**Keywords:** Retina, Gliosis, Müller Glia, Neuroprotection, In vitro, Retinopathies, Anti-VEGF, Bioengineering

## Abstract

Progressive and irreversible vision loss in mature and aging adults creates a health and economic burden, worldwide. Despite the advancements of many contemporary therapies to restore vision, few approaches have considered the innate benefits of gliosis, the endogenous processes of retinal repair that precede vision loss. Retinal gliosis is fundamentally driven by Müller glia (MG) and is characterized by three primary cellular mechanisms: hypertrophy, proliferation, and migration. In early stages of gliosis, these processes have neuroprotective potential to halt the progression of disease and encourage synaptic activity among neurons. Later stages, however, can lead to glial scarring, which is a hallmark of disease progression and blindness. As a result, the neuroprotective abilities of MG have remained incompletely explored and poorly integrated into current treatment regimens.

Bioengineering study of the intrinsic behaviors of MG hold promise to exploit glial reparative ability, while repressing neurodisruptive MG responses. In particular, recent in vitro systems have become primary models to analyze individual gliotic processes and provide a steppingstone for in vivo strategies. This review highlights recent studies of MG gliosis seeking to harness MG neuroprotective ability for regeneration using contemporary biotechnologies. We emphasize the importance of studying gliosis as a reparative mechanism, rather than disregarding it as an unfortunate clinical prognosis in diseased retina.

## 1 Introduction

**Fig. 1.**
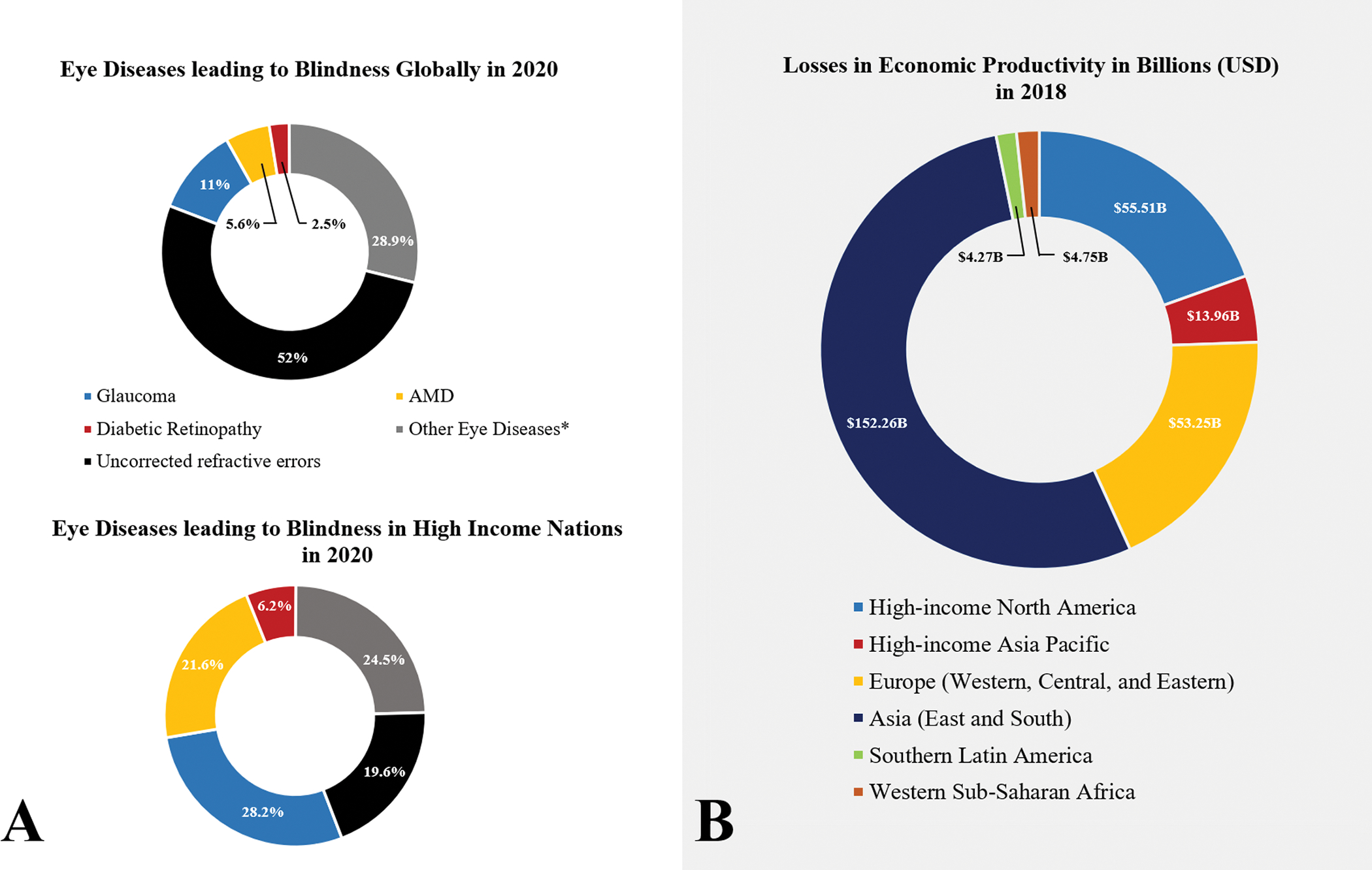
Health and Economic Burden of Retinopathies, worldwide. **(A)** Prevalence of blindness in adults (50 years and older) in 2020 as a consequence of common retinopathies: glaucoma, diabetic retinopathy, age-related macular degeneration (AMD), and other retinopathies (e.g. ocular trauma and corneal diseases). Uncorrected refractive errors are defined as eye conditions that cause blur vision, such as myopia, hyperopia, and cataracts. These conditions can be corrected via prescription glasses, and/or surgery **(B)** Losses in economic productivity worldwide in billions (USD).

Rising levels of vision loss among adults in the United States (US) will severely impact the lifestyle, employment opportunities, and health of millions of Americans in the current decade [1]. Additionally, visual Impairment imposes a significant socio-economic burden upon local and global communities, as associated health care costs in the US have recently exceeded $139 billion, annually, compared to $105.4 billion in China and $68.5 billion in the European Union [2–4]. Likewise, losses in economic productivity have made a significant impact in the gross domestic product (GDP) worldwide, as seen in Fig 1. [5]

Many adults will lose vision as a result of dysfunction in the retina, a photosensitive tissue that lines the inner portion of the eye and converts photons into visual information. Over 9 million Americans currently endure progressive vision loss from common retinopathies, such as age-related macular degeneration (AMD), glaucoma, and diabetic retinopathy (DR). These diseases are caused by degenerated retinal neurons, elevated intraocular pressure, and abnormal blood vessel growth, respectively (Reviewed in here). Many contemporary therapies seek to restore vision by stimulating the regeneration of retinal neurons using pharmaceutical treatments, neural implants, and/or surgical interventions [6–10]. However, few studies have explored retinal repair by harnessing the innate, neuroprotective responses of Müller glia (MG) cells, which undergo complex processes of gliosis to ameliorate retinal damage.

### 1.1 Retinal Gliosis

Gliosis is an evolutionary response to injury characterized by a myriad of molecular and cellular responses to initiate repair. In mammals, retinal gliosis offers a brief period of neuroprotective mechanisms to foster regeneration, before the natural wound healing process leads to scar formation. Particularly, MG undergo gliosis, defined as a series of endogenous repair responses that increase the cellular metabolic rate to protect and support neurons, as well as to isolate damaged cells upon retinal injury [11, 12]. MG are the first responders to retinal injury and respond accordingly, depending on the severity of the insult, to ultimately determine the level of visual impairment. During initial, or short-term, gliosis, MG become reactive by undergoing hypertrophic changes in morphology, radial extension of cellular processes, and proliferation. These reactive behaviors are also accompanied by the upregulation of neurotrophic factors, cytokines and enhanced homeostatic regulation in MG to ensure neuronal survival and communication [13]. However, prolonged gliotic responses can produce excessive MG proliferation, which displaces adjacent neurons to disrupt synaptic networks, curtail transmission of photonic signaling, and impede retinal regeneration [14]. Chronic gliosis can also lead to retinal scarring, which is a primary factor in progressive visual impairment and subsequent vision loss. Enriched understanding of the underlying mechanisms of MG responses will enable transformative approaches to manipulate gliotic processes to prevent extensive retinal scarring while encouraging neuronal communication.

### 1.2 Current In Vivo Approaches to Restore Vision

Reactive gliosis of MG is an endogenous response to retinal insults, irrespective of the underlying retinopathy. Development of the retinal glial scar is well-studied and is a primary cause of vision loss. However, current treatments can only decelerate the progression of retinopathy by alleviating indirect factors that exacerbate MG gliotic response, such as aberrant angiogenesis and increased intraocular pressure. The glial scar impacts a number of promising therapies, including cell replacement, delivery of viral vectors, pharmaceutical treatments, and electro-modulation. Specifically, glial scarring hinders the migration and integration of exogenous cells in the tissue that intend to regenerate damaged tissue [15]. Likewise, delivery of viral vectors or pharmacological agents to the degenerated area is hindered due to the physical and chemical barrier posed by glial cells [16, 17]. Lastly, the effectiveness of neural probes and other eletro-modulation therapies have been challenged by the intrinsic response of the body to encapsulate and isolate a foreign body led by glial cells [18, 19].

#### 1.2.1 Laser Treatment and Anti-angiogenic Factors

Pan-retinal photocoagulation (PRP) is a popular procedure for patients diagnosed with late-state or proliferative DR, wet AMD, and neovascular glaucoma. The procedure uses fundus imaging to identify abnormal blood vessels, which are then laser-cauterized to reduce their expansion. Conventional laser treatments are also accompanied by anti-angiogenic drugs, such as Avastin®, Lucentis®, and Eylea®, to restrain the growth of new blood vessels and encourage vessel regression [20, 21]. Although this treatment regimen decelerates progressive retinal degeneration, it does not restore lost vision. This is because glial scarring permanently disrupts synaptic communication and leads to hypoxic regions that induce the recurrence of leaky blood vessels as retinopathy worsens. In these cases, PRP has been rendered insufficient, while reports of diminished visual acuity (VA) after anti-angiogenic drugs have questioned the efficacy of long-term pharmacological treatment [22]. In fact, the outcome of anti-angiogenic drug clinical trials, such as the Comparison of Age-related Macular Degeneration Treatments Trials (CATT), has been further analyzed years post-treatment to assess the long-term effects of these drugs in patients. The CATT reported that the incidence of retinal scarring was higher in patients undergoing antiangiogenic treatment than the scarring incidence post-treatment [23]. Interestingly, though these drugs have demonstrated significant efficacy in reducing aberrant angiogenesis and slowing the progression of vision loss, the anti-angiogenics contribute to higher prevalence of retinal scarring. Future studies must assess the effects of these agents in MG, as the inhibition of VEGF leads to metabolic disruption in MG that can result in gliosis or apoptosis [24, 25]. Thus, accelerating retinal scar formation during treatment [26]. Table 1 summarizes the prescription outcome of treatment in first world modernized nations, as well as the advantages and disadvantages of anti-VEGF treatments [27–29]

#### 1.2.2 Genetic Engineering

Retinal dysfunction that arises from genetic mutation(s), such as retinitis pigmentosa and Leber congenital amaurosis, has been examined using gene editing tools, including transcription activator-like effector nucleases (TALENs) [30], Zinc-finger nucleases [31], and the most recent clustered regularly interspaced short palindromic repeats (CRISPR/Cas9) technology [32, 33]. These tools have allowed researchers to identify mutations in retinal neurons and glia to, both, edit the dysfunctional genetic code and replace it with functional sequences [34]. However, stable delivery of genetic material is difficult because it must remain intact and able to reach the nuclei of targeted cells. As a result, adeno-associated virus (AAV) vectors, liposomes, and nanoparticles have been recently used as cargo transporters to effectively deliver molecules across the cellular membrane [35]. Recent exciting progress has used AAV vectors to restore protein levels in rodent and human retina models of retinitis pigmentosa (RP), an inherited disease characterized by the loss of photoreceptors (PRs) [36, 37]. Likewise, AVV therapy has demonstrated its safety in clinical trials of RP, although its efficacy in restoring vision is yet to be assessed. A recent study showed that 50% of a patient cohort that was administered AAV therapy to deliver a corrected version of the hMERTK gene, developed visual impairment after treatment, and improvement in VA was lost in 60% of the remaining patients 2 years post-operation [38]. Moreover, in vivo studies have demonstrated that AAV therapy is most effective in retinopathies where the inner limiting membrane (ILM) is removed or becomes compromised due to degeneration to allow vectors to cross into the neuro-retina and transduce multiple cell-types [39]. However, removal or weakening of the ILM may lead to macular dysfunction due to synaptic disruption, as the end feet of MG are key to the membrane’s structure and promote axonal activity [40, 41]. Likewise, conventional subretinal injection, which traverses the ILM into the subretinal space, may not be a viable option in advanced cases of retinal degeneration as retinal detachment may occur due to the force exerted by the injection [42]. Therefore, AAV therapy delivery still faces challenges, such as MG activation and exacerbation of glial scarring [43]. Despite the efficacy of this therapy in delivering correct copies of genes to restore cellular function, the exacerbation of gliosis poses a key barrier in the success of this therapy. Hence, decreasing MG reactivity prior to AAV delivery would result in a better outcome for the patient to slow the progression of disease and restore vision.

Other recent developments in regenerative medicine have explored techniques of MG cellular transdifferentiation, a form of direct metaplasia that skips de-differentiation stages in between cell lineages [44]. Research groups have sought to convert mammalian MG into PRs with the help of gene editing and molecule agonists by forcibly activating pathways, such as the Sonic hedgehog (SHH) signaling pathway [45, 46]. The results of these experiments have demonstrated that MG retain an innate ability to dedifferentiate into other cells like PRs, yet these cells need to be guided through the cell cycle for this phenomena to occur. Moreover, MG have been shown to transdifferentiate into functional neurons, in vivo, via AAV gene delivery, although lineage is restricted to limited types of neurons [47–49]. While transdifferentiation offers an innovative approach to convert MG into neuronal replacement cells, such a therapy will not achieve retinal regeneration independently. This limitation is because MG transdifferentiation requires the coupling of methodologies to induce changes in genes expressed by MG, as well as a delivery system for therapy, in situ, complicating this approach. While many groups have begun to deconvolve the regenerative mechanisms of MG [50–52] from species where this process is spontaneous, regenerative strategies that harness endogenous MG neuroprotective responses remain understudied (reviewed here [53]). Technical approaches able to examine the mechanistic underpinnings of MG behaviors will provide invaluable knowledge for the development of therapies to combat vision loss.

More recently, low-current electrical stimulation was used to upregulate proliferation in MG, as well as increasing MG expression of neuronal markers and production of neurotrophic factors[54]. Specifically, electric stimulation has upregulated the production of endogenous Brain-Derived Neurotrophic Factor (BDNF) in cultured MG [55], supporting claims of previous studies that showed a correlation between electrical stimulation and upregulation of ciliary neurotrophic factor (CTNF) and BDNF. In those works, the latter increased the survival of damaged photoreceptors in vitro but increased the number of gliotic MG with enlarged soma [56]. This exciting development demonstrates that electrical stimulation has potential benefits for retinal repair, including direct transdifferentiation of MG and increased secretion of neurotrophic factors. However, this methodology needs further study and characterization, particularly for the induction of MG into a reactive state and subsequent proliferation, which is a hallmark of neuroglia scarring.

#### 1.2.3 Cell Replacement Therapy

One promising contemporary therapy is stem cell replacement, where a variety of stem-like cells derived from embryos [57], somatic cells [58], induced pluripotent stem cells [59], and de-differentiated adult cells [51] are transplanted into host retina to replace damaged neurons. Here, transplanted cells must survive insertion, navigate within damaged neural tissue to achieve appropriate cellular positioning, and synapse with endogenous cells to reactivate the neural networks of vision. While each of these complex processes provides significant challenges to restore vision, all remain in need of enriched mechanistic understanding [60]. Moreover, a major obstacle to the functional integration of transplanted cells is overcoming the physical barrier imposed by the glial scar [61]. Despite advances in regulated stem cell differentiation and development of transplantable biomaterials, an incomplete understanding and control of glial scarring during endogenous response to insult has continued to actively hinder the functional integration of replacement cells [62].

### 1.3 In Vitro Approaches to Examine Gliosis in the Retina

Despite remarkable advancements to restore vision, MG gliosis poses the major obstacle for current biomedical approaches to repair and regenerate damaged tissue. Upon retinopathy, a myriad of regulatory processes are activated to ensure proper functioning of the retina; thus, altering the behavior of each cell type, and the overall synergistic relationship among them. Specifically, the complexity of the in vivo environment, including its biochemical composition, cellular interconnectivity, and feedback mechanisms, are major challenges to understand the response of MG to a range of external stimuli that leads to gliosis. Hence, conditions of retinopathy have been extrapolated to an in vitro environment to permit investigating fundamental cellular responses to controlled changes in the surrounding biochemical and cellular environment. In vitro platforms enable researchers to deconvolve complex environments into elementary, but representative models of organs and tissues. Contemporary microscale platforms enable the pursuit of hypotheses on the intrinsic behavior of native and transplantable retinal cells, without external factors that may alter cell response to stimuli.

In vitro technologies, including cell cultures [63–65], microfluidic devices [66, 67], organoids [68–70], and others [71, 72], can be applied to study the morphology, migration, proliferation, and/or cell interconnectivity of retinal cells with high precision (Reviewed in [73]). Physiological changes in retinal cells can also be studied as a function of time within these same models, which is not possible in vivo. MG reactivity can be particularly well-characterized via microscale assays [74] to determine the parameters that induce their neuroprotective responses, as well as identify biochemical cues that exacerbate gliosis. Moreover, in vitro assays can be tailored to mimic retinal models of disease at specific stages of degeneration [75, 76], which coupled with their adaptability to imaging methods such as microscopy, facilitate highly detailed and quantitative study of cellular behavior. Although in vivo studies provide the most translatable results to the clinical setting, research that couples these data with in vitro studies can transformatively enrich contemporary understanding of the parameters that mediate changes in retinal cell behavior, particularly the factors that induce gliosis and how they are regulated. Herein, this body of work highlights the need to incorporate therapies that aim to enhance the neuroprotective mechanisms of MG, while preventing the onset of chronic scarring to repair the retina. The collaboration of in vitro studies and the in vivo validation of these hypotheses will advance current regenerative therapies to break paradigms on vision restoration. Refer to Table 2 for a detailed summary of the advantages and disadvantages of in vivo therapies to restore vision, as well as the use of in vitro approaches to characterize MG gliosis [77–81]

## 2 Retinal Anatomy

The retina is a multi-laminated structure populated by an interconnected network of millions of neurons with the ability to convert photons into electrochemical signals that are transmitted to produce images of objects in the brain. Likewise, glial cells of the retina, including Müller glia (MG), astrocytes, and microglia, help support phototransduction via regulation of ions, cytokines and trophic factor production. Particularly, the radial nature of macroglia, such as MG and astrocytes, allows them to expand their processes to provide mechanical support and biochemical regulation of ions to guide neuronal synapses [82]. On the other hand, microglia play a key role during development via synaptic pruning in the developing retina, as well as in immune surveillance through the production of pro-inflammatory factors and clearing of cellular debris upon injury [83].

**Fig. 2.**
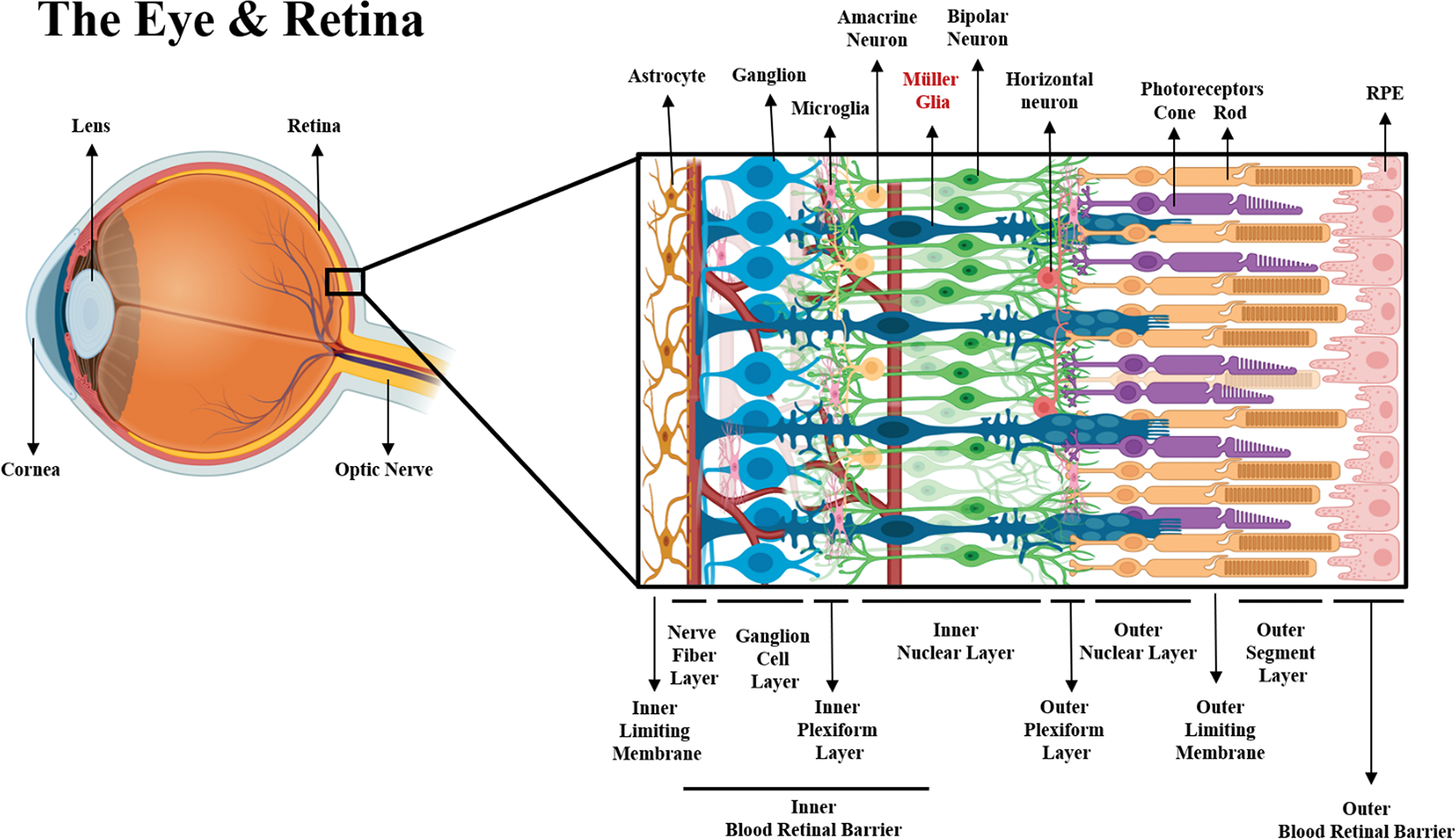
Schematic of the Eye and Retinal Structure. Neuronal and neuroglia components of the retina within their respective layers. From the posterior to anterior side of the retina (left to right), the end-feet of Müller glia and astrocytes form the inner limiting membrane, along with extracellular matrices such as fibronectin collagen, laminin, proteoglycans and hyaluronans [124]. The axons of ganglion cells bundle to form the optic nerve at the nerve fiber layer (NFL), while their dendrites communicate with both bipolar and amacrine cells. There are approximately 18 different types of ganglion cells that reside in the ganglion cell layer (GCL) [125], forming synaptic connections with bipolar cells via graded potentials [126]. BCs are divided into two major classes depending on their sensitivity to glutamate released by photoreceptors. In the presence of glutamate, ON-BCs will depolarize to continue synaptic activity, while OFF-BCs hyperpolarize to attenuate synapses [127]. The communication of GCs and BCs is also regulated by ACs via gap junctions, which actively release both GABA and glycine to modulate the biochemical communication between neurons at the inner plexiform layer [128]. Despite the inhibitory signaling mediation of most ACs, a different type of ACs (Starburst ACs) are known to be the only cholinergic neurons in the retina that work as excitatory regulators of neural signaling [129]. In the inner nuclear layer the bodies of most BCs, ACs, MG and horizontal cells (HCs) are found. Likewise, dense vascularization is present in the region between the NFL and the INL to supply neurons and glia with oxygen and nutrients, forming the inner blood retinal barrier (iBRB) [130]. Dendrites of BCs connect with photoreceptors at the outer plexiform layer via glutamatergic synapses, which are modulated by GABAergic signaling from HCs. The proactive communication of HCs via gap junctions serve to enhance or decrease signaling from both types of photoreceptors, cones and rods [131]. The outer nuclear layer hosts the bodies of photoreceptors, as well as processes of MG that extend radially to aid in the metabolic function of these neurons, forming a mechanical barrier known as the outer limiting membrane, which provides stability to the retina and helps to selectively permeate molecules coming from the subretinal space [109]. There are three types of cone photoreceptors, each sensitive to a specific wavelength of light (red, blue, and green), whereas one single type of rod photoreceptor. Nonetheless, rods outnumber cones in a 10:1 ratio in the mammalian retina and they are more sensitive to light [132]. The outer segments of photoreceptors initiate phototransduction via a biochemical process involving the protein rhodopsin contained in membrane organelles called “discs” that are replaced everyday [133]. The vast molecular activity involving cellular waste uptake and nutrient transport to the photoreceptors is regulated by the retinal pigmented epithelium cells [134], which via tight junctions form the outer blood retinal barrier that regulates the flux of molecules from the choriocapillaris into the neural retina [135].

The human retina lines the back of the posterior portion of the eye and is shaped like a crescent with varying thickness. The fovea, the region of highest visual acuity (VA), ranges in thickness between 150–200μm (thinnest area of the retina), whereas the retinal periphery can reach up to 320μm (thickest area of the retina) [84–86]. Incident light refracted into the eye is first absorbed by rod and cone photoreceptors (PRs), located in the retinal outer nuclear layer (ONL). Photoreceptors synapse with retinal secondary neurons (i.e. horizontal and bipolar cells) located within the inner nuclear layer (INL), while MG regulate waste removal and the biochemical recycling of neurotransmitters critical to phototransduction, such as glutamate [87, 88]. MG are particularly sensitive to neurotransmitters, such as acetylcholine, which triggers calcium transients inside these cells to modulate neuronal activity and vasomotor function [89, 90]. In development, neuronal activity is primarily regulated by γ-aminobutyric acid (GABA), preceding glutamate as the primary excitatory molecule [91]. In later stages, glutamate becomes the dominant neurotransmitter released by amacrine cells and ganglion cells (GCs), where the latter are found in the ganglion cell layer (GCL) [92, 93] as seen in Fig. 2.

The GCL includes MG processes that surround the body of GCs for optimal waste removal, nutrition support and structural strength all throughout the layer [94–96]. Axons of GCs extend and bundle into nerve fibers to constitute the optic nerve, the direct connection from the eye to the brain. The optic nerve transmits electrical information to the visual cortex of the brain to form images of objects [97]. The optic nerve requires a large supply of nutrients, primarily provided by retinal blood vessels. The large biochemical activity of the optic nerve is also regulated by astrocytes and MG whose end feet provide aid to the NFL to ensure balance of nutrients and waste removal. Moreover, the metabolic regulation of the retina is aided by the inner blood retinal barrier (iBRB), a selective physiological barrier that supplies the retina with oxygen and nutrients. The iBRB is made of endothelial cells (ECs) wrapped by pericytes and covered by the foot processes of glial cells like ACs and MG. MG regulate the flux of neurotransmitters, ions, and nutrients to maintain the homeostasis in the retinal parenchyma [98]. Yet, transport across the iBRB is regulated by two major factors: the formation of tight junctions among ECs and gap junction communication between ECs and MG. Tight junctions are formed by protein complexes such as zonula occludens 1 (ZO-1) and claudin, fusing ECs together, forcing selected molecules to move from the blood into the neural retina mostly via transcellular transport. Gap junctions proteins like connexin 43 (Cx43) and connexin 45 (Cx45) are expressed in both MG and ECs, forming intracellular channels for the regulation of molecule transport [99, 100]. Likewise, the expression of these gap junctions has been demonstrated to influence the expression of the glutamate/aspartate transporter (GLAST), which is expressed in MG to maintain low levels of circulating glutamate in the retina. Research has demonstrated that Cx43 also plays a fundamental role in the survival of MG and ECs, as downregulation of Cx43 in response to hyperglycemia leads to MG, pericyte, and ECs apoptosis, ultimately threatening the physiological ability of the iBRB [101, 102].

### 2.1 Müller Glia and Neurons

MG communication with neurons and glia is fundamental for healthy functioning of the retina. For instance, specialized gap junctions in vertebrates allow the passage of molecules and ions such as Ca2+ and K+ to facilitate synapse signaling [103, 104]. Additionally, MG regulate the uptake of neurotransmitters like glutamate [105], as well as neurotransmitter inhibitors such as GABA [87] and glycine [106] to maintain the physiology of the retina. Although neurons are able to recycle glutamate and uptake GABA, MG contribute to the efficiency of these processes, as accumulation of glutamate in the retina leads to neuronal death [96]. Interestingly, communication between MG and other cells independently of mammalian class occurs through paracrine signaling, modulated by receptors located at the surface of their membrane [107]. Paracrine signaling in MG has also been demonstrated to play a fundamental role in phototransduction and communication with other glial cells, such as astrocytes and microglia, due to the ability of MG to propagate Ca2+ waves initiated by nearby neurons [103]. Additionally, research has also discovered that purinergic signaling involving Ca2+ waves in MG allow them to start their own signaling through the depolarization of Ca2+ stores in their endoplasmic reticulum, postulating an independence to modulate synapses without extrinsic influence [108]. Despite the importance of paracrine signaling between neurons and glia, there is a gap in the understanding on how Müller glia can enhance synaptic signaling between neurons when the retina becomes compromised.

Tight junctions like Zonula occludents-1 (ZO-1) form between MG and photoreceptors at the OLM, constituting a mechanical barrier to provide stability to the retina [109]. Tight junctions also work as a selective biochemical barrier that impedes the diffusion of macromolecules into the retinal parenchyma, further regulating the transport of cytokines, water, and proteins coming from the retinal epithelium [110]. Yet, the neuron and MG interaction is perhaps most exemplified by retinal homeostatic regulation, where they work in concert to regulate phototoxicity [96]. PRs release glutamate as they depolarize in the absence of light, while some BCs and GCs release it in the presence of light to maintain continuous retinal synapse. Likewise, in the posterior side of the retina, MG and GCs interconnectivity plays a fundamental role in signal transduction. The ensheathing of GC soma by MG processes has demonstrated to modulate and direct axonal growth, particularly during the post-natal phase (P3), as MG release glycoproteins such as Tenascin-C and Laminin, which help in axon taxis [111]. However, the release of laminin is significantly reduced in the adult retina, limiting the aid to encourage axonal growth post-injury [41]. In disease, the communication between neurons and MG is altered, characterized by changes in gap junction and tight junction expression, as well as phenotypic change in MG soma that disrupts their intrinsic connectivity. In neovascular retinopathies like DR, the expression of gap junctions is decreased, slowing the metabolic activity and inducing MG reactivity that contributes to scar formation [112]. Additionally, these changes induce changes in the viscoelastic properties of MG via hypertrophy, discouraging neurite growth and reducing neuronal plasticity [113]. Interestingly, MG in vertebrates communicate differently with neurons in different species, particularly upon retinal damage. In zebra fish and frogs, MG are able to transdifferentiate into neurons to reinstate neuronal connectivity [114, 115], also reviewed in [116]. These changes occur as a result of a combination of genetic, as well as epigenetic changes driven by evolution (Reviewed in [117, 118]). Unfortunately, in mammals MG are unable to naturally transdifferentiate or de-differentiate into neurons to restore neuronal communication. However, research has demonstrated that by upregulating genes such as ASCL1 [119] and increasing the concentration of heparin-binding EGF (HBEGF) [120], MG are able to decrease their reactivity and de-differentiate into a progenitor stem-cell like state. Hence, new therapies can aim to preserve the native communication between neurons and MG to maintain cell-to-cell connectivity post-injury, as this is one of the major challenges for current regenerative therapies in gliotic retina.

### 2.2 Müller Glia and Other Retinal Glia

Glia-glia interaction has been of interest due to their combinatory response to retinal injury. Both retinal macroglia, ACs and MG respond to injury through an upregulation of glial fibrillary acid protein (GFAP) and Vimentin, followed by cellular hypertrophy which leads to increasing mechanical stiffness in the retina. Studies have shown that though both ACs and MG react via gliosis upon injury, their mechanisms of action differ. For instance, after initial hypertrophy, MG proliferate to fill any breaks caused by damage to the retinal structure, whereas ACs create a glia scar bordering the injury [41]. Nonetheless, both macroglia work synergistically in response to retinal damage, communicating through paracrine signaling, often through ATP mediated communication [103]. On the other hand, MG and microglia play a synergistic role in both healthy and diseased retina. In healthy retina, microglia are located largely in the synaptic layers and GCL of the retina, surveilling for foreign molecules and/or pathogens, while aiding axonal directionality through synaptic pruning [121]. Upon injury, the increasing paracrine communication of MG with both neurons and glia, activate microglia to initiate an inflammatory response, characterized by the upregulation of pro-inflammatory factors such as: interleukin-1-beta (IL-1β), interleukin-6 (IL-6), and inducible nitric oxide synthase (iNOS) [122]. Active microglia also promote the production of adhesion molecules like vascular cell adhesion molecule-1 (VCAM-1) and intercellular adhesion molecules (ICAM-1) in MG, increasing the contact attachments for microglia to adhere, as it has been shown that MG processes serve as scaffolds for microglia to migrate. Lastly, activation of microglia also is known to drive the upregulation of cytokines that promote MG migration and further extension of their processes. The activation of glial cells upon injury has been depicted as the decisive point of no return, where any possibility for tissue regeneration is abandoned as scarring would permanently seal the injured area. However, activation of glial cells carry neuroprotective processes that are paramount for regeneration and tissue remodeling. There is indeed a period of time from the activation of glial cells to the onset of scarring that needs to be further explored to accelerate retinal repair. For instance, ACs and MG have the ability to go into hypertrophy to strengthen the retinal tissue from inside and along the NFL to avoid retinal detachment. Research has demonstrated that higher expression intermediate filaments such as Nestin and GFAP is essential to stabilize and strengthen the NFL in response to mechanical damage [123]. Likewise, MG are able to direct microglia via extension of their processes towards damaged areas of the retina to phagocyte debris, which is fundamental for retinal repair and synaptic remodeling.

## 3 Role of Müller Glia in The Diseased Retina

Müller glia (MG) along with astrocytes are the most predominant neuroglia of the retina, yet MG are argued to be the only macroglia endogenous to the neuroretina while astrocytes are thought to migrate from the optic nerve [41, 117]. MG processes at the OLM stabilize the outer segments of PRs, which do not possess junctional complexes, but rather connect to retinal pigmented epithelium cells (RPE) through weak adhesive junctions [134, 136]. At the posterior side of the retina, MG foot processes play an important role in establishing the inner BRB, selectively regulating the flux of toxins and nutrients that travel in the blood. In addition, the soma of MG is present throughout the other retinal layers, where processes extend radially to maintain the homeostasis of the interstitial space and regulate the metabolic function of neurons [137].

Mammalian MG respond to injury by increasing their metabolic function, including the production of neurotrophins like nerve growth factor (NGF) and BDNF to promote neuronal survival, upregulation of antioxidants like glutathione to prevent oxidative stress during hypoxia, and phagocytosis of cellular debris [138]. These responses are considered neuroprotective to halt further damage in the retinal tissue as a consequence of disease or injury. However, depending on the severity of the retinopathy, the response by MG could be more aggressive, exacerbating retinal injury, changing from a passive approach to a reactive one.

Reactive MG readily extend their processes towards the damaged area and increase the secretion of pro-inflammatory factors such as the transforming growth factor beta (TGFβ) superfamily, downregulate proteins like cellular retinaldehyde binding protein (CRALBP), which leads to the activation of molecular pathways involved in the accelerated proliferation and hypertrophy of MG [14, 139–141]. Uncontrolled MG proliferation and hypertrophy alters MG mechanical properties of MG, resulting in stiffer cellular soma and increased cellular processes. These in turn reduce synaptic plasticity between neurons, impair neurite growth, and restrict neuron-to-neuron connectivity [142]. Ultimately, reactive gliosis leads to scar formation within and outside of the retina, displacing nearby neurons and altering the layer structure of the retina. The glial scar rapidly fills the injured area with the soma of glial cells, which upregulate the production of proteins, like fibronectin and collagen, to create a physical and biochemical barrier to protect the remaining tissue [143, 144]. Formation of a glial scar impedes neuronal regeneration and tissue remodeling to restore function. For a more thorough description of fundamental mechanisms of disease retina and glial scar formation, the following reviews are recommended for additional information (Reviewed in [11, 145]).

### 3.1 Müller Glia in Diabetic retinopathy

Diabetic retinopathy (DR) is the result of a variety of complications including aberrant angiogenesis, edema and reactive gliosis, as a consequence of complications of diabetes mellitus, characterized by hyperglycemia and hypoxia in the retina [146]. DR is estimated to affect 16 million Americans by 2050 and is currently incurable [147].

Although DR is the result of multiple etiologies, symptoms emerge as the disease progresses [148]. The most aggressive and degenerative stage of this disease is called proliferative diabetic retinopathy (PDR), which is characterized by the formation of leaky blood vessels that lead to hemorrhage and edema. Particularly, hyperglycemia increases MG reactivity due to overwhelming production of glutamate, which is not completely recycled by these cells and leads to neurotoxicity [98]. MG in response to high neurotoxin levels releases endogenous vascular endothelial growth factor (VEGF) to encourage angiogenesis and nutrient supply to the affected area [149]. However, the dramatic increase of VEGF rushes the formation of immature capillaries, often leaky blood vessels, increasing the permeability to toxins and waste coming from the blood. Furthermore, MG-derived VEGF-a has been linked to the rapid loss of tight junction proteins such as occluding and zonula occludens in endothelial cells, accelerating the degeneration of the iBRB [150]. Nonetheless, the initial expression of VEGF-a can be considered neuroprotective as it promotes neuronal survival and encourages the production of interleukin-6 (IL-6) in ECs, which has demonstrated to be protective of MG under hyperglycemic conditions. In fact, IL-6 has recently become a target for neovascular retinopathies, since it increases the metabolic activity of MG at early stages of disease [98]. Likewise, MG has been characterized to undergo hypertrophy in DR, which can strengthen the mechanical support to the retinal tissue to avoid retinal layer separation, as the formation of new blood vessels disrupt the cellular organization in the retina [151].

### 3.2 Müller Glia in Wet Age-related macular degeneration (AMD)

AMD is a degenerative disease of the retina affecting the macula, causing loss in the center field of vision [152]. AMD is characterized by the disruption of the retinal structure, leading to the death of PRs and weakening of the retinal pigmented epithelium layer [153].

Aging is a major factor influencing the symptoms of AMD, which include the accumulation of proteins and fatty acids, known as drusen, within the retinal layers as a result of slower physiological activity of MG [154]. Slower recycling of glutamate often leads to lower levels of glutathione, an antioxidant produced by MG that prevents neuronal damage, mainly released to counteract free oxygen radicals present during high phototransduction activity [87]. Reduced levels of glutathione in vertebrates have been demonstrated to accelerate the development of age related retinopathies like AMD [155]. However, further complications involving genetics and the onset of other retinopathies that develop concurrently may influence the progression of AMD [156]. Dry AMD is the most common type of this retinopathy, involving the accumulation of drusen, small piles of waste product and fat, that disrupts the structure of retinal layers. Further complications of dry AMD eventually leads to wet AMD, a more aggressive form of this disease characterized by leaky blood vessels, causing edema, particularly under the macula, affecting the central vision of the retina [157]. Drusen activates MG by inducing the upregulation of intermediate filaments, causing hypertrophy. MG radially extend their processes away from the outer nuclear layer reaching the outer limiting membrane, weaving around the bodies of photoreceptors. In larger accumulations of drusen, MG has been observed to wrap around these depositions and form a glial scar [158]. Nonetheless, the engulfing of drusen can be accessed as neuroprotective, as this prevents these protein and waste deposits from accumulating, further causing neuronal death.

### 3.3 Müller glia in Neovascular Glaucoma

Neovascular glaucoma (NVG) is a complication of open angle glaucoma, characterized by the formation of new blood vessels that obstruct the flow of aqueous humor, leading to increased intraocular pressure, and ischemia. This aggravated form of glaucoma is often the result of ischemic retinopathies, such as central vein retinal occlusion or central artery retinal occlusion [159]. The pathology of NVG is accompanied by an increasing intraocular pressure (IOP) that compresses the retina between the vitreous humor and the choroid, leading to cell death, breaking of capillaries, and consequently damaging the overall structure of the tissue. Likewise, increasing intraocular pressure often results from slower drainage of the aqueous humor at the front of the eye. The accumulation of fluid increases the pressure within the eye and onto the retina [160]. MG in response to the increasing pressure become reactive and start producing neuroprotective molecules like leukemia inhibitory factor (LIF), a signaling molecule that enhances glia to glia communication to induce a collective response against injury. However, high concentrations of LIF induces excitotoxicity in GCs [161]. Astrocytes and microglia also play an important role in the pathogenesis of NVG, primarily driven by the upregulation of pro-inflammatory factors expressed by microglia. Upon pathogenesis, microglia upregulate IL-1β, which induce the upregulation of chemokine ligands such as Cxcl1 and Cxcl10 in MG, further enhancing their reactivity and gliotic response [162]. MG also interact with astrocytes to create a fibrotic barrier at the NFL via hypertrophy and enlargement of their foot processes. The increasing IOP activates astrocytes, which subsequently release pro-inflammatory factors such as Tumor Necrosis Factor alpha (TNF-α) that further increases the reactivity of MG within the retina [163]. Additionally, glaucoma disease induces the upregulation of other molecules like endothelin2 (Edn2), which is a signaling molecule present during photoreceptor and ganglion cell death to facilitate the communication between glia to neurons. Yet, increasing levels of Edn2 induce MG reactivity, exacerbating their gliotic reaction [164, 165].

## 4 Examining the Neuroprotective Response of MG

The neuroprotective ability of MG was not considered in warm-blooded vertebrates until 20 years ago [166]. The advancement of technologies such as gene sequencing, microscopy, and bioinformatics have enabled MG characterization that has contributed to the contemporary understanding of MG role(s) in retinal function and repair [167–169]. Novel applications of micro-technologies have more recently provided a new medium to study MG in vitro, specifically playing a key role in examining the individual cell responses of cells at the molecular level with high precision. Particularly, Microdevices have provided a unique platform to parse the complex gliotic phenomena into quantifiable changes of cellular reactivity. Changes in MG behavior including hypertrophy, proliferation, migration, and cellular interconnectivity have been studied in vitro to analyze the intrinsic response of these cells to changes in their environment important during gliosis and retinal repair.

Culture dishes have been used to make significant contributions to assess phenotypic changes of MG in response to stimuli over time [170–172]. Most recently, bioengineering groups have built upon these in vitro systems to model the extracellular landscape using micropatterned substrates [173–175] based on the different stages of gliosis. Likewise, the chemotactic ability of MG has been quantified using transwell assays, which have the ability to retain concentration gradients of cytokines overtime [176–178]. In addition, complex engineering systems such as microfluidic devices have served as tunable platforms to study migratory patterns of MG under specific biochemical conditions, as well as providing a medium to study cellular interconnectivity through the use of microchannels in compartmentalized chambers.

**Fig. 3.**
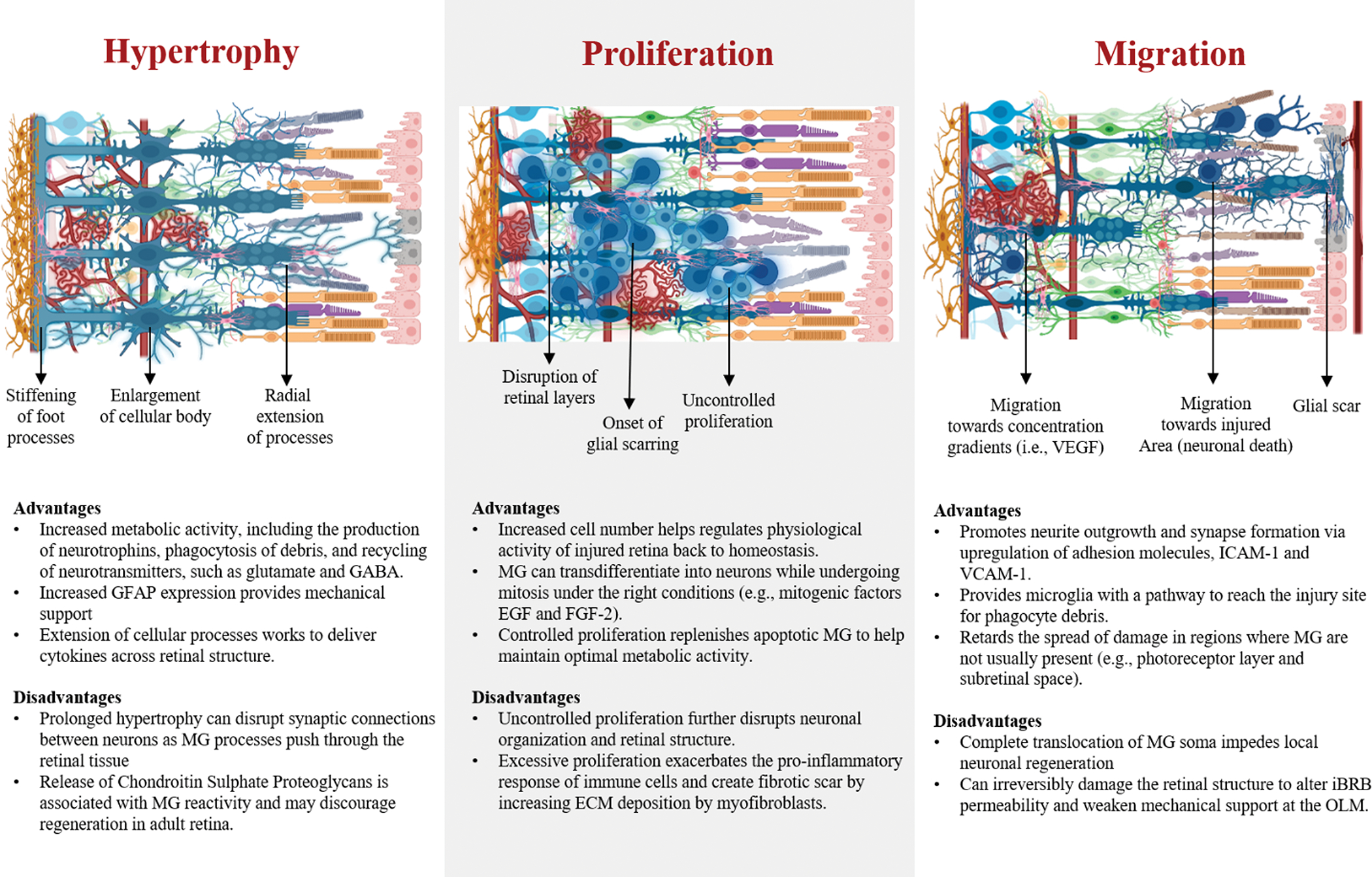
Advantages and Disadvantages of Primary Gliotic Behaviors of Müller Glia. Effects of progressive hypertrophy, proliferation, and migration in diseased retina.

3D in vitro models of the retina like organoids and hydrogels, have been essential to characterize MG behaviors such as proliferation within a more relevant physiological level. However, the turning point when these neuroprotective behaviors turn into a neurodisruptive action that leads to scar formation remains poorly understood. Therefore, further examination using in vitro devices to characterize the individual neuroprotective response of MG is paramount to design therapies that can be implemented in vivo. The advantage of these platforms relies on the tunability of the environment to study specific cues of gliosis in MG. Depending on the concentration of cytokines, extracellular matrices, and other stimuli, stages of gliosis can be recreated to study the neuroprotective behaviors that are inherently present in these cells. Likewise, characterization of the factors that turn the reparative action of MG into chronic gliosis are key to determine the type of therapy that would suit best a patient. Hence, further examination of the three major gliotic behaviors of MG: hypertrophy, proliferation, and migration, (Fig. 3) would fill in the current knowledge gap to harness the endogenous neuroprotective potential of MG while avoiding the adverse effects that cause permanent vision impairment. The authors recommend these reviews for a more comprehensive review on the particular impact of in vitro technology, such as culture dishes [63], microfluidics [179], and organoids [180] in retinal research.

### 4.1 Hypertrophy in MG

Hypertrophy is one of the major reactive behaviors of MG, characterized by the enlargement of their soma, increased expression of GFAP, and upregulation of intermediate filaments like vimentin. Hypertrophy has been observed to occur first than proliferation in MG, in contrast to other macroglia like astrocytes, which seem to go under hyperplasia before hypertrophy upon injury [181, 182]. Particularly, GFAP expression in MG is recognized as one of the principal markers of glial reactivity, linked to changes in cellular phenotype, which may alter the mechanical properties of MG by increasing cellular stiffness, known to reduce neuron plasticity [183]. However, reduction of neuron plasticity might be dependent on the extracellular matrix remodeling around the hypertrophic bodies of MG rather than MG soma, as increased concentrations of ECMs such as laminin and collagen may alter both neuronal survival and proliferation [184]. In fact, explants of rodent retina have shown that GFAP upregulation leads to extension of macroglia processes, which seem to stimulate neurite outgrowth in ganglion cells by working as scaffolds. Likewise, GFAP expression in MG has been associated to help with the retention of the glutamate transporter (GLAST) in their membranes, paramount for the recycling of upregulated glutamate produced by neurons in stress [185]. Although hypertrophy has been associated with the negative impact of gliosis, this process is also fundamental to increase the physiological activity of MG to maintain the retinal homeostasis. Acute hypertrophy may be beneficial for the retina due to the role of MG in providing mechanical support to the retinal layers, as well as upregulating the production of neurotrophins and recycling of waste. Furthermore, MG are able to downregulate the expression of GFAP as it is not ubiquitously expressed as it is in astrocytes [137]. MG by assuming a short-term expression of GFAP and hypertrophy may enhance their metabolic capability to aid neurons during acute disease, and then assume a quiescent state once the retinal tissue reaches homeostasis. Hence, reactivity of MG is required to stabilize the retinal tissue upon injury. Specifically, molecular processes associated with hypertrophy provide great benefit to halt the breakdown of the retinal layers, as well as to supply neurons with neurotrophins needed to maintain synaptic activity. Modulating the hypertrophic response of MG to remain transient should be the scope of current therapies that aim to induce regeneration, as this process is reversible and does not cause permanent damage at acute stage.

### 4.2 Proliferation in MG

Proliferation is considered one of the hallmarks of reactive gliosis and the onset of retinal scarring. However, the rate of glia proliferation depends on the severity of the retinal insult, as lower degree of injury results in a more conservative, non-proliferative behavior in MG. In cases of significant retinal damage, MG proliferate and rapidly seal the injured area, followed by collagen deposition from myofibroblasts, a scar is formed [186]. Nonetheless, the outcome of MG proliferation is diverse among species, which ultimately determines the ability for vision restoration. Studies have demonstrated that reactive mammalian MG, in contrast to MG of amphibians and teleost fishes, display different levels of gene expression and enzyme activity during the cell cycle, which causes the failure to de-differentiate into a progenitor state to initiate regeneration [187]. Instead, mammalian MG rapidly transition from the interphase into the mitotic cell cycle, which results in cellular division [188]. Some reports have attributed that the natural downregulation of the protein p27kip1, a cyclin enzyme inhibitor, at precise timepoints of the cell cycle leads to proliferation of MG and upregulation of GFAP [189, 190]. However, the inhibition of cyclin proteins like cyclin D1, a key modulator of stem cell properties in MG during the cell cycle, may inhibit the regenerative capability of mammalian MG [191]. Nonetheless, numerous research groups have manipulated the expression of key genes in mammalian MG or introduced specific factors to their environment to circumvent their limited regenerative ability, resulting in positive outcomes, among those the de-differentiation of MG into progenitor stem-cell like cells to drive regeneration [46, 47, 192]. Other approaches have aimed to identify growth factors and molecules that may influence MG proliferation, which could help to modulate this behavior by inhibition or stimulation of specific cellular pathways. For instance, research has shown that proliferation of MG can be stopped via molecular pathway inhibition of the ERK1/2 and PI3K/AKT pathways, which results in a moderate proliferation of MG that is beneficial to upregulate the production of neurotrophic factors, [193]. Overall, these findings are key to develop therapies that lead to in situ regeneration, as well as supporting current therapies like stem-cell transplantation to become more effective, as scar formation constitutes a major roadblock for stem cell integration. Hence, further investigation that aims to characterize the mechanisms that influence mammalian MG proliferation and their ability to dedifferentiate into progenitor cells warrants more investigation.

### 4.3 Migration in MG

Migration of MG is a critical step in the development of gliosis, mostly observed in degenerated retinas of low cellular density as a result of injury. In the event of vast cellular death, MG are able to push through the retinal layers migrating several tenths of microns. For instance, mammalian MG migration has been characterized in vivo, where their nuclei traveled an average distance of 30 microns after laser-induced injury towards the lesion site [194]. Likewise MG migration has been studied in models of the zebrafish, where their nuclei have been observed to migrate from the INL to the ONL in response to photoreceptor death [137]. Furthermore, migration of MG has been identified to occur in the opposite direction from the INL to the ILM in idiopathic cases of epiretinal membranes (EMs), a fibrous tissue on the surface of the retina, adjacent to the GCL [195, 196]. Recent data has shown that the formation of EMs enables complete MG translocation from the INL to the retina’s surface, altering the synaptic communication between neurons, and compromising the integrity of the ILM [40].

The migration of MG has been particularly characterized in response to the upregulation of growth factors such as EGF and VEGF [13, 177, 197]. Concentration gradients of VEGF have demonstrated to increase the chemotactic response of MG in vitro, which correlated to the increased concentration of VEGF in later stages of neovascular retinopathies in vitro (21484851, 24412518). As neuronal death is extensive, MG are able to migrate towards the site of injury to aid in the recycling of cellular debris and glial scar formation to prevent further damage. Although the migration of MG may further disrupt the retinal architecture as neurons are displaced during the migration process, this is only possible if there is low neuronal density to allow MG translocation. In the event, of acute retinopathy, MG migration is hindered by tightly packed neuronal and glial bodies. Yet, processes associated to MG movement such as the expression of adhesion molecules may be beneficial to stimulate directed neurite growth and migration of microglia to support the natural immune response of the body [122].

Researchers have recently identified the upregulation of cellular adhesion molecules (CAMs), including the intercellular adhesion molecule 1 (ICAM-1) and the vascular cell adhesion molecule 1 (VCAM-1), in both primary mammalian MG and in a human cell-line of MG (MIO-M1) [198]. The activation of microglia and their migration towards the site of injury is extremely important, as microglia actively phagocyte dead neurons as well as stimulate the release of trophic factors, like glial cell line-derived neurotrophic factor (GDNF) and leukemia inhibitory factor (LIF), both playing important roles in neuronal survival [122, 199]. In the same way, the expression of CAMs have been demonstrated to promote neuronal growth and support synapse formation [200]. Undoubtedly, the neuroprotection of MG goes beyond their direct homeostatic communication with neurons, as their activity indirectly stimulates other cells, e.g. microglia, to help ameliorate the effects of the retinal insult. Perhaps, new reparative therapies at early stage of disease may look into modulating the glial reactivity to remain as a transient neuroprotective effect, characterized by the expression of CAMs and neurotrophic factors, rather than a long-lasting process that may result in disruptive effects in neuronal organization.

## 5 Gliosis in The Retina

Gliosis has been regarded as the key biomarker for irreversible vision loss and current research has been focused on detering this process from happening in the first place. Correspondingly, examining gliosis as a potential reparative strategy has been challenged by the lack of knowledge of its neuroprotective behaviors. However, in case of retinal damage, glial cells are necessary to restore function, and halting their innate ability to restore homeostasis is counterproductive. We can harness the neuroprotective abilities of MG to restore vision by assessing the benefits and drawbacks of gliosis at different stages of disease.

### 5.1 Gliosis: The Bad

Gliosis poses a physical barrier for the advancement of regenerative therapies in the retina. Current treatments against neovascular retinopathies have primarily focused on discouraging aberrant angiogenesis, yet gliosis has not been addressed despite being the main factor in irreversible vision loss. Scar formation driven by reactive MG remodels the retinal environment via several processes. For instance, MG increase the secretion of matrix metalloproteinases (MMPs) that degrade extracellular matrices like collagen, facilitating processes such as proliferation and hypertrophy, which displaces nearby neurons, and disrupts the compact architecture of the retinal layers. Additionally, upregulation of proinflammatory factors like TNF-a and IL-1B by macrophages, encourage MG proliferation and chemokinesis in early stages, yet overexposure to these factors lead to glia apoptosis, dysregulating the retinal homeostasis. Likewise, these reactive behaviors pose a barrier for newer regenerative approaches to succeed. For example, stem cell therapy is one of the most promising regenerative strategies in the central nervous system, effective in slowing down the progression of disease [201]. However, gliosis challenges this approach in several ways. First, retinal stem-cell transplantation triggers a pro-inflammatory response due to their foreign origin, which activates macrophages and microglia, and subsequently MG. Research has demonstrated that upon stem-cell introduction in retina, there is an immediate hypertrophic reaction by MG, characterized by the upregulation of intermediate filaments that lead to increased tractional forces and eventual retinal folding [62]. On the other hand, when a retinal scar is already present, stem cells cannot migrate into this region nor integrate with the host tissue, rendering this therapy ineffective.

Retinal prosthetics have introduced a new alternative to restore vision in patients with late stage of retinal degeneration, where photoreceptors are not able to initiate phototransduction [202, 203]. Prosthetics require the implantation of electrodes on the epiretinal surface or in the subretinal space to initiate electrical signaling that is sent to the brain. Multiple devices have either used a stimulator that activates the electrodes or have relied on natural light to induce signaling. However, the implantation of these devices usually activates glial cells, particularly on the epiretinal surface, which leads to increased stiffness in glial cells and lower plasticity in neurons [204, 205]. Likewise, the insertion of an electrode array in the subretinal area requires space within the retinal tissue, which causes activation of MG and the exacerbation gliosis, or in cases of late degeneration, an extensive scarred area would limit the space for an electrode array implantation [206, 207].

Lastly, pharmacological approaches have served to reduce the progression of neovascular retinopathies by inhibiting aberrant angiogenesis, which may reduce exacerbation of the pathology. In fact, pharmacological agents like anti-VEGF drugs have demonstrated to decrease the rate of vision loss, and in some cases improve the visual acuity of patients [208, 209]. Nonetheless, anti-VEGF drugs could carry out detrimental changes in the retinal physiology long-term, such as geographic atrophy, choroidal thinning, and inducing apoptosis in neurons and glia by disrupting VEGF-mediated survival pathways. [24, 210]. Likewise, research from clinical trials have recorded the increased presence of geographic atrophy (GA) [211], a common effect in aged retina involving death of neurons and retinal epithelium cells, in patients undergoing anti-VEGF treatment, raising the concern of a possible link between these therapies and exacerbation of gliosis.

### 5.2 Gliosis: The Good

Overall, biomedical approaches have aimed to reduce the progression of vision loss through MG transdifferentiation into retinal progenitors, completely halting gliosis, and/or reducing available cytokines known to induce reactivity in glia. However, the underlying problem, chronic gliosis, which leads to irreversible vision loss and prevents the success of existent therapies, remains overlooked. For instance, new approaches that aim to transdifferentiate MG into retinal progenitors, mimicking the endogenous regenerative response present in other animals (fish or frogs), have been developed in hope to restore vision. However, the mammalian retinal physiology in contrast to lower vertebrates, requires a more active role of MG during injury due to the lower metabolic rate in humans [212], unhealthy dietary patterns [213], and differences in retinal cell density [214]. Hence, completely halting gliosis may be counterproductive and likely to exacerbate pathology in the long run. Harnessing the neuroprotective ability of MG while reducing the onset of chronic gliosis may be the best approach to restore vision in humans, while improving the efficacy of current regenerative therapies, such as cell replacement therapy. Likewise, there is a need for new therapies, methodologies, and techniques that can be concurrently implemented to counteract the disruptive behaviors of MG in retinopathy.Certainly, the complete halt of MG activity would further exacerbate retinal pathology, but therapies that focus on enhancing MG neuroprotective ability, while discouraging neurodisruptive reactivity, would render the best results to decelerate progression of disease and broaden a path towards retinal regeneration.

Furthermore, the three different gliotic behaviors are accompanied by protective or reparative processes that can be beneficial for the retinal environment.

Acute glial reactivity, characterized by transient hypertrophy [215] and often accompanied with recycling of cytokines, production of neurotrophic factors [139], phagocytosis of cellular debris, enhanced communication with other glial cells [216], and increased mechanical support to the retina [217], is the pinnacle of MG reparative ability in mammalian retina. Proliferation and migration of MG are more complex behaviors that are present in later stages of disease aimed to contain the damage from further extending to other parts of the retina. Although these two processes carry out a neurodisruptive activity by remodeling the architecture of the retina and inhibiting neuronal synapses, the containment of an ongoing problem such as accumulation of drusen, edema, and an exacerbated pro-inflammatory response is equally important. Partial vision loss may result from the proliferation and migration of MG, but their inability to stop the damage may lead to a more rapid degeneration and complete vision loss.

Modulation of glial activity can be also implemented as part of prophylactic therapies that aim to treat chronic retinopathies that require the active, enhanced metabolic regulation of MG, while curbing its neurodisruptive behaviors. For this reason, gaining knowledge on the specific mechanisms that drive gliosis and how we can manipulate them is the stepping stone to meet these goals. Hence, in vitro platforms become a key instrument to assess the response of MG to different environments that simulate disease. In vitro platforms such as well-plates, microfluidic devices, transwell assays, etc. permit the isolation of MG from the multiple factors that play a role in neovascular retinopathies, enabling the characterization of transient and long-term reactive behaviors linked to a specific stimulus. Indeed, in vitro platforms can provide additional information on the elementary mechanisms of MG repair, which may be key to develop new approaches to halt retinal degeneration and encourage regeneration.

## 6 Future Research and Limitations

Despite contemporary technological and therapeutic advances to treat neovascular retinopathies, glial scarring remains a primary challenge to the efficacy of these approaches. As a result, development of prophylactic therapies to halt chronic gliosis in early stages of disease is an effective methodology to prevent irreversible vision loss. Absence of retinal scarring would 1) prevent thickening of the NFL that often progresses into retinal detachment [123], 2) facilitate migration and integration of transplanted cells to foster regeneration in cell-replacement therapy [218], 3) enhance delivery of gene therapy across the ILM by reducing morphological alterations from chronic gliosis [42], [43], and 4) conserve integrity of the iBRB by reducing macroglia reactivity [11].

Given increasing vision loss in patients suffering from neovascular retinopathies, new alternatives are needed to prevent chronic gliosis that may result in scarring. For instance, new developments in the areas of genetic engineering and drug therapy have started addressing gliosis to prevent retinal scarring.

### 6.1 Genetic Engineering Perspective

Understanding the molecular changes that induce MG gliosis is paramount to block the late-stage, neurodisruptive behaviors that cause glial scarring while preserving early stage neuroprotective abilities. Particularly, several groups have recently identified key genes that modulate the different stages of MG gliosis using microRNAs. [219] The inhibition of genes such as Atf3, Egr2, Maff, and Gadd45b demonstrated a significant reduction of gliotic responses in the injured retina of adult mice, while preserving retinal structure. This advance is key as a stepping stone to explore new avenues for the development of targeted therapies that modulate MG response(s) to injury. Additionally, inhibition of the Notch-1 signaling pathway has been of particular interest to attenuate gliosis due to its effect on upregulating pro-inflammatory factors, such as TGF-B [220]. Recent study has demonstrated that inhibition of Notch-1 signaling via intraretinal injection of RO4929097 significantly reduced MG-induced gliosis, while also preventing the overexpression of ECMs involved in scarring and remodeling [221]. Gene therapy is an efficient and targeted therapy for early stages of neovascular retinopathy. However, major challenges are present in the method of gene delivery and the overall impact of this therapy to reduce chronic gliosis. AAV has been selected as the most viable vehicle of gene delivery due to its lower immunogenicity. However, epiretinal scar formation poses a major challenge for vector delivery, reducing its load into the neuroretina [39]. Likewise, additional challenges for AAV vectors remain in its low payload to deliver longer sequences of genes [222]. This represents a challenge for gene therapy since most genetic modification that leads to meaningful changes, at the structural and metabolic level, require longer coding base pairs. Furthermore, the etiology of neovascular retinopathies is largely polygenic, which would require additional combinatory treatments to address the overall pathology of the injured retina [223].

### 6.2 Drug Therapy Perspective

The pathology of neovascular retinopathies include a myriad of physiological changes in the retina that remain unaddressed. For instance, hypercholesterolemia, a condition characterized by high levels of cholesterol, is highly present in patients suffering from DR, AMD, and glaucoma. Research in rabbits has demonstrated the use of statins (drugs currently used to reduce cholesterol) had a significant impact on reducing chronic retinal gliosis when administered in low dosages for a period of 8 months [224]. Astrocytes and MG displayed GFAP positive bodies, but lower fiber bundles and scar-like structures in the treated group. Likewise, a study [225] in diabetic patients treated with statins reported lower levels of pro-inflammatory factors and molecules in the vitreous humor, including TGFB-1, VEGF, and MMP-9. The prolonged upregulation of these markers particularly contribute to the breakdown of the iBRB, activate glial cells, and induce fibrosis in the retina. The study illustrated that statins significantly reduced the formation of fibrotic tissue in DR patients. Similarly, another group recently reported that the use of low-dose statins was associated with 28% lower risk of re-vitrectomy in a diabetic cohort of adults in Finland [226]. These independent results suggest that the use of current pharmacology to treat distinct ailments of neovascular disease may have positive impacts that reduce scar formation. Combinatory drug therapies to reduce chronic gliotic responses and aberrant angiogenesis, thereby, show promise towards decelerating the progression of neovascular retinopathies and promoting tissue repair. However, drug therapy continues to face the significant challenge of chronic gliosis. Despite numerous drug therapies to reduce the progression of vision loss in neovascular retinopathies, their efficacy is vastly limited by the stage of retinopathy. In late stages of retinal disease, geographic atrophy, characterized by irreversible changes in the retinal structure and dense scarring, impede the uniform diffusion of drugs into the neuroretina [227]. Additionally, the effect of these therapies in advanced stages of disease will have minimal effect on restoring vision [228]. Hence, early detection of retinal insults and a proactive drug therapy plan will provide greater benefits to prevent structural damage and restore function.

### 6.3 Antioxidant Therapy Perspective

Neovascular retinopathies are affected by the accumulation of reactive oxygen species (ROS), chemically reactive molecules that are generated as a sub-product of cellular metabolism [229] due to hypoxia, cell death, and tissue homeostatic imbalance. Numerous research groups have explored the use of natural bioactive compounds such as antioxidants to reduce the accumulation of ROS and improve neuronal survival in retinopathy. Extracts of ginkgo biloba (GB), one of the oldest tree species on earth, have been used for thousands of years to reduce inflammation, working as a neuroprotectant [230]. Specifically, clinical trials on glaucoma patients who received 80mg twice a day of GB extracts for over 4 years significantly decelerated the damage of their visual field with respect to control [231]. Likewise, a case study on a glaucoma patient showed an improvement in VA after 11 months of GB treatments [232].

Additionally, anthocyanins, a group of natural substances found in bilberry (Vaccinium myrtillus L.), have been studied due to their promising effects in vision health (reviewed in [233]). Particularly, the effects of anthocyanins significantly improved mean changes of visual field in glaucoma patients after 2 years of treatment, following the Mean Deviation Humphrey visual field index [234]. Moreover, another antioxidant that has gathered interest is Aloin, a bioactive compound in aloe vera with antiinflammatory properties [235]. Recently, the neuroprotective effects of Aloin were evaluated in a hepatic-retinopathy rat model, where the administration of Aloin for 5 days significantly reduced the swelling of MG, preventing chronic gliosis and disruption to the retinal structure [236]. However, the effects of Aloin in the retina still need to be elucidated, particularly their effects after long-term administration. Overall, these results demonstrate the benefit of antioxidants against retinopathies when administered for at least a year, restricting their use to a preventative approach. Besides facing the common challenge of chronic gliosis to irreversibly damage the retina, natural antioxidants need to be further studied to validate their efficacy as a prophylactic therapy. Nonetheless, current results show promising evidence of these bioactive compounds to improve vision health.

## 7 Conclusion

Neovascular retinopathies are the most aggressive type of eye diseases leading to progressive vision loss, leading to a major burden on the health of millions of adults worldwide. While research and clinical therapies have worked together to develop treatments to ameliorate the negative impact of disease, there is no current cure for these retinopathies. The majority of vision research, such as cell transplantation, genetic engineering, pharmaceutical treatment, and others, have focused on restoring the neuronal component of the retina, as well as preventing angiogenesis to foster regeneration. However, these therapeutics face a common roadblock that impedes any advance towards retinal repair, which is glial scarring that is often wrongly defined as gliosis. Fundamentally, gliosis encompasses the activation of glial cells with the ability to repair, while scarring is the physiological response of the body to wound healing as a result of chronic gliosis. Despite the adverse effects of scarring, the onset of gliosis is accompanied by neuroprotective processes that are essential for tissue repair. Yet, deep understanding of the factors that modulate the gliotic response in MG require further investigation. Different approaches have demonstrated to be successful, both in vitro and in vivo, to characterize MG behavior. Nonetheless, a step beyond is required to implement this knowledge into therapeutics that can harness the protective ability of MG and prevent chronic scarring. Furthermore, manipulation of gliotic behaviors would provide a window for other current and emergent approaches to eliminate their major obstacle to restore vision. Likewise, the modulation of gliosis could serve as a therapy for ongoing retinopathies that require a higher level of metabolic function in the retina. Ultimately, gliosis should not be assessed as an unfortunate event of human evolution, but rather a tool that can be used for tissue repair.

## Figures and Tables

**Table 1.** Analysis of Anti-VEGF therapies Effectiveness in The United States & United Kingdom.

**Treatment**	**Estimated Number of Prescriptions in 2015**	**Response to Treatment**	**Change in BCVA LogMAR after 1 year**	**Prevalence of Scar (%) CATT**

**Lucentis® (Ranibizumab)**	• US = 697,412 • UK = 367,608	64.4%	0.187	48%
**Eylea® (Aflibercept)**	• US = 870,843 • UK = 216,708	73%	0.2	No Data
**Avastin® (Bevacizumab)**	• US = 1,147,432 • UK = 16,920	59.2%	0.16	53%



**Therapy/Approach**	**Advantages**	**Disadvantages**

**Anti-VEGF Injections**	• Hinders the progression of aberrant neovascularization. • Effective in improving visual acuity (VA) in early stages of retinopathy or at good VA baseline. • Procedure is performed in an ambulatory environment. • In the US, Medicare Part B covers 80% of Eylea® injections and 100% of Lucentis®, once the annual deductible has been paid. • A cheaper but less effective treatment, Avastin®, is prescribed off-label for a fraction of the price.	• Costly procedure. A two-year treatment (Aflibercept and Lucentis) averages 20,000 USD/year, or ∼ 30% of the US median household income in 2020. • VA improvement largely depends on baseline values. Low VA results will render mild to little VA improvement. • Retinal scarring is not addressed, and progressive gliosis can still lead to blindness. • Treatments require several injections, usually every 4 weeks during the first 3 to 4 months. Each session requires 1 or 2 days for the patient to recover (soreness and/or blurred vision).

Data of FDA approved and actively prescribed therapies against neovascular retinopathies in developed nations (These nations were selected as per data availability). Treatment outcomes of the three major anti-VEGF drugs prescribed in the US and UK. The number of yearly prescriptions in the UK were estimated based on the total number of prescriptions of January 2015, and multiplied by 12. The yearly US data was readily available. Response to treatment after 1 year was defined as “… the absence of lesion activity (that is, disappearance of the features of fluid in any of the macular tissue compartments), or the reduction in fluid by up to 75% of the baseline values”. Changes in BCVA (Best Corrected Visual Acuity) of the LogMAR chart were calculated based on the improvement from baseline values after 1 year of treatment. A value of 0 represents 20/20 visual acuity while a value of 1 represents 20/200. Prevalence of scarring are cited from the Comparisons of Age-Related Macular Degeneration Treatments Trials (CATT).

**Table 2.** Summary of In Vivo and In Vitro Approaches for Retinal Treatment

**In Vivo Therapy**	**Advantages**	**Disadvantages**

**Anti-VEGF treatments**	• Only current FDA-approved therapy against neovascular retinopathies • Effective in improving visual acuity when administered at early stage of pathological neovascularization	• Short-effect in visual acuity improvement. Neovascularization is recurrent post-treatment. • Can induce rapid development of glial scarring during treatment
**Cell Replacement**	• Stem cells can differentiate into damaged neural cells • Low risk of immune rejection when using autologous cells	• Significant challenges with synaptic integration in adult retinal host • Potential of tumor formation
**Genetic Engineering**	• Targeted gene editing to restore function in defective cells • Wide selection of vectors for highly specific therapy with low immune reaction	• Success of therapy is largely dependent on the availability of functional cells (i.e., neurons). • Insufficient in late stages of retinopathy. • Rising bioethical issues with types of therapy (i.e., CRISPR).



**In Vitro Research**	**Advantages**	**Disadvantages**

**Culture Dishes (2D)**	• Inexpensive, straightforward set-up, and wide availability of literature and protocols. • Control of concentration and exposure time for stimuli • Compatible multiple imaging techniques (brightfield, fluorescent, and electron microscopy)	• Does not resemble in vivo geometry or physiology • Kinetics of the medium are often neglected • Isolation of cells from host tissue into a 2D culture can affect their phenotype and function
**Microfluidic Systems**	• Highly tunable systems that can be modified to resemble key characteristics of in vivo geometry and/or physiology • Real-time study of cell behaviors using conventional imaging.	• Requires training and specialized tools for microfabrication • Large number of microfluidic systems feature a 2D environment
**Organoid Models (3D)**	• In vivo-like environment (3D) with preserved cellular phenotype and metabolic function • Conventional cell-to-cell communication	• Variable level of oxygen from the surface to the core of the organoid • Expensive and difficult to characterize using conventional imaging.
